# Induction of circulating phospholipase A_2_ by intravenous administration of recombinant human tumour necrosis factor

**DOI:** 10.1155/S0962935192000358

**Published:** 1992

**Authors:** Waldemar Pruzanski, Matthew L. Sherman, Donald W. Kufe, Peter Vadas

**Affiliations:** 1 Inflammation Research Group University of Toronto The Wellesley Hospital 160 Wellesley St. East, Ontario Toronto M4Y 1J3 Canada; 2 Laboratory of Clinical Pharmacology Dana-Farber Cancer Institute Harvard Medical School Boston MA USA

## Abstract

We have examined the effects of intravenous infusion of recombinant human tumour necrosis factor (rh-TNF) on serum activity of phospholipase A_2_ (PLA_2_) in patients with malignancies. Nine patients received a 24 h continuous intravenous infusion ranging from 1.0 × 10^5^ U/m^2^ to 3.0 × 10^5^ U/m^2^; 14 patients received a 5 day continuous intravenous infusion ranging from 0.5 × 10^5^ U/m^2^/day to 3.0 10^5^ U/m^2^/day. Twenty one of 23 patients responded with marked increases in serum PLA_2_ activity that were detectable 3 h after the beginning of the rh-TNF infusion and reached maximum levels at 18 h with a mean increase of 16.2-fold. In patients receiving a 5 day rh-TNF infusion, the highest levels of PLA_2_ were observed after the first day of infusion. Serum PLA_2_ activity declined continuously to 2.9-fold above baseline at the end of the infusion. A significant correlation was noted between the dose of infused rh-TNF and the maximum increase in PLA_2_ activity. To our knowledge, this is the first time that an association between intravenous TNF administration and induction of circulating PLA_2_ in man has been established.


Mediators of Inflammation, 1, 235–240 (1992)

### Induction of circulating phospholipase $A_2$ by intravenous administration of recombinant human tumour necrosis factor

Waldemar Pruzanski,[1,CA] Matthew L. Sherman,[2] Donald W. Kufe,[2] and Peter Vadas[1]

[1]Inflammation Research Group, University of Toronto, The Wellesley Hospital, 160 Wellesley St. East, Toronto, Ontario, Canada M4Y 1J3;
[2]Laboratory of Clinical Pharmacology, Dana-Farber Cancer Institute, Harvard Medical School, Boston, MA, USA

[CA] Corresponding Author

**WE have examined the effects of intravenous infusion of recombinant human tumour necrosis factor (rh-TNF) on serum activity of phospholipase $A_2$ ($PLA_2$) in patients with malignancies. Nine patients received a 24 h continuous intravenous infusion ranging from $1.0 \times 10^5$ U/m$^2$ to $3.0 \times 10^5$ U/m$^2$; 14 patients received a 5 day continuous intravenous infusion ranging from $0.5 \times 10^5$ U/m$^2$/day to $3.0 \times 10^5$ U/m$^2$/day. Twenty one of 23 patients responded with marked increases in serum $PLA_2$ activity that were detectable 3 h after the beginning of the rh-TNF infusion and reached maximum levels at 18 h with a mean increase of 16.2-fold. In patients receiving a 5 day rh-TNF infusion, the highest levels of $PLA_2$ were observed after the first day of infusion. Serum $PLA_2$ activity declined continuously to 2.9-fold above baseline at the end of the infusion. A significant correlation was noted between the dose of infused rh-TNF and the maximum increase in $PLA_2$ activity. To our knowledge, this is the first time that an association between intravenous TNF administration and induction of circulating $PLA_2$ in man has been established.**

**Key words:** Cancer patients, Phospholipase $A_2$, Recombinant human tumour necrosis factor

### Introduction

Phospholipase $A_2$ is a lipolytic enzyme that hydrolyses membrane associated phospholipids of mammalian cells and initiates the arachidonic acid cascade.[1] Several of its end product eicosanoids have well known proinflammatory activity.[2] Recent studies have demonstrated that phospholipase $A_2$ ($PLA_2$) is secreted extracellularly in inflammatory sites including synovial fluids in inflammatory arthritides,[3] peritoneal fluid in peritonitis,[4] and sera in septic shock.[5,6] $PLA_2$ injected into skin,[7] joints[8] or paws[9] of experimental animals elicits a time and dose-dependent inflammatory reaction. We have previously shown that intravenous injection of endotoxin in experimental animals[5] or human volunteers,[10] was associated with a marked increase in circulating $PLA_2$ activity. Furthermore, intravenous infusion of $PLA_2$ reproduces many features of endotoxaemia.[5]

Bacterial and toxic insults lead to the rapid synthesis and release of cytokines including IL-1 and TNF.[11–14] We recently demonstrated that these two cytokines markedly enhance the synthesis and extracellular release of $PLA_2$ from cultured mammalian cells.[15] Infusion of TNF in man elicits clinical and haemodynamic manifestations similar to those caused by endotoxin.[16,17] Since infusion of $PLA_2$ leads to similar manifestations,[5] it was of significant interest to determine whether recombinant human TNF (rh-TNF), given intravenously, would be followed by intravascular secretion of $PLA_2$ *in vivo*. The finding that TNF infusions, without prestimulation with endotoxin, cause increase in circulating $PLA_2$ would reinforce the link between proximal cytokine response and $PLA_2$ activity, and may provide insights into the mechanisms of the proinflammatory activity of TNF. Such findings may also indicate that TNF has an impact on activation of the $PLA_2$-initiated arachidonic acid cascade. The present study demonstrates that intravenous administration of TNF in humans leads to a rapid and marked increase in circulating $PLA_2$ activity.

### Materials and Methods

*Patient selection and therapy:* Twenty-three patients with cancer (Tables 1 and 2) received intravenous infusion of human recombinant tumour necrosis factor (rh-TNF) (Asahi Chemical Industry Company of America, New York, NY). Nine patients (group I) received a 24 h continuous intravenous infusion,[16] and 14 patients (group II) received a 5 day continuous intravenous infusion.[17] In the first group the rh-TNF dose varied from $1.0 \times 10^5$ U/m$^2$/day to $3.0 \times 10^5$ U/m$^2$/day. In the second group the daily dose ranged from $0.5 \times 10^5$ U/m$^2$/day to $3.0 \times 10^5$ U/m$^2$/day. The first group also

© 1992 Rapid Communications of Oxford Ltd received etoposide (Bristol-Myers Oncology, Wallingford, CT), 80 mg.m$^{-2}$ by continuous intravenous infusion for 3 days, starting 24 h prior to rh-TNF infusion. Both groups were pretreated with indomethacin 50 mg on the evening before and q.i.d. thereafter for 24 h. All patients were hospitalized and monitored as described.[16–18] Sodium chloride (0.9%) was infused at 150 ml.h$^{1}$ for 24 h prior to rh-TNF infusion. rh-TNF was diluted in the same buffer and infused at a constant rate (150–300 ml/day). In all patients, previous chemotherapy and/or radiotherapy was discontinued at least 3 weeks prior to rh-TNF infusion.

*Drug formulation:* The rh-TNF used for this study had a specific activity of 2.3 × 10$^{6}$ U/mg protein. One unit was defined as the amount required to lyse 50% of L-M cells in a 48 h assay. The preparation of TNF used in this study contained less than 100 pg of endotoxin per mg protein, as tested by the Limulus lysate assay.

*Enzyme assay:* Phospholipase $A_2$ assay was performed as described, using autoclaved *Escherichia coli* $K_{12}C_{600}$ labelled with [$^{14}$C]oleic acid as the substrate.[19] Assays were performed in substrate excess, using 2.8 × 10$^{8}$ *E. coli* per assay, corresponding to 5.6 nmol of phospholipid with a specific activity of 4120 cpm/nmol. In conditions of substrate excess, the rate of substrate hydrolysis was linear with reaction times up to 30 min, over a five-fold range of enzyme concentration. One unit of $PLA_2$ activity is defined as the amount of enzyme activity that hydrolyses 56 pmol of *E. coli* phospholipid in 30 min. Activity of serum $PLA_2$ after rh-TNF infusion was tested at different pH and calcium concentrations as described previously.[19,20] The effect of neutralizing polyclonal antibody (NPA) against rh-$PLA_2$ (lot 207, Biogen, Cambridge, MA) on rh-TNF-induced endogenous $PLA_2$ was tested by incubating NPA with $PLA_2$-containing sera for 60 min at room temperature, and testing the mixture for residual $PLA_2$ activity. The direct effect of rh-TNF on $PLA_2$ activity *in vitro* was tested using *E. coli* phospholipid substrate. rh-TNF in concentrations of up to 1000 U/ml were preincubated with $PLA_2$ for 30 min at room temperature prior to addition of substrate.

The reference range for normal serum $PLA_2$ ($n = 143$) is 149 ± 69 (SD) U/ml with a range of 40–365 U/ml. Serum samples from patients infused with rh-TNF were coded and assayed for $PLA_2$ activity without any knowledge regarding the relationship of the sample to the time, dose or the nature of infusion. The results were simultaneously exchanged and analysed in two centres.

*Statistical analysis:* Statistical analysis was performed by standard tests including correlation coefficient and Student's *t*-test.

### Results

Group I consisted of three men and six women ranging in age from 25 to 73 y (mean 52.2 y). The primary tumours of these patients are summarized in Table 1. Metastases, mainly to the liver and lung, were present in all patients. One patient had a past history of cancer of the breast, one had tuberous sclerosis and one had mild, chronic pancreatitis. None had fever or infection preceding the rh-TNF infusion. White blood cell count varied from 5 to 11 × 10$^{12}$ / l , with a normal differential count. All patients had normal creatinine.

Before rh-TNF infusion six of the nine patients had normal serum $PLA_2$. In three patients, two with renal cell carcinoma and one with cancer of the colon, baseline $PLA_2$ was elevated ranging from 727 U/ml$^{-1}$ to 2297 U / ml . Seven of nine patients responded to rh-TNF infusion with marked increases in $PLA_2$ (Table 1). Increase in $PLA_2$ activity was evident at 3 h after beginning the rh-TNF administration, and lasted for the entire period of infusion. Maximal $PLA_2$ activity (16.2-fold) was observed at 18 h of infusion (Figure 1). Levels of $PLA_2$ remained elevated (1510–38274 U/ml) in all three patients in whom the serum was tested 24 h after terminating the infusion.

In group II, there were seven men and seven women ranging in age from 37 to 70 y (mean 55.4 y). The diagnoses are summarized in Table 2. Metastases were detected in twelve of 14 patients. No past diseases known to influence $PLA_2$ activity, such as pancreatitis, rheumatoid arthritis, infection or fever were documented. All patients had normal renal function (creatinine <1.2 mg/dl). Peripheral blood leukocyte count ranged from 4 to 14 × 10$^{12}$/l with normal differential counts.

Ten of 14 patients had normal serum $PLA_2$ activity prior to rh-TNF infusion (Table 2). In four patients, initial serum $PLA_2$ levels were elevated ranging from 604 U/ml to 6397 U/ml. All 14 patients demonstrated an increase in $PLA_2$ activity after initiation of the rh-TNF infusion (Table 2). The activity was highest after the first day of infusion (13-fold) and it gradually declined to 2.9 times higher than baseline after 5 days of infusion, and two times higher than baseline 24 h after termination of rh-TNF infusion (Fig. 2).

In both groups, there was a significant correlation between the daily dose of rh-TNF infused and the maximum increase in $PLA_2$ activity ($p < 0.05$) (Fig. 3).

$PLA_2$ in the sera of rh-TNF infused patients was calcium dependent, with optimal activity at 5 mM and was completely inactivated by 2 mM EDTA. The pH optimum was 7.5. Polyclonal antibodies against human group II $PLA_2$ completely neu-

**Table 1.** Clinical/laboratory profile of patients who received continuous 24 h intravenous infusion of rh-TNF

| No. | Age | Sex | Diagnosis* | Dose of rh-TNF U/m$^2$ | PLA$_2$ U/ml pre-TNF | PLA$_2$ U/ml peak during TNF infusion |
|---|---|---|---|---|---|---|
| 1 | 53 | F | Renal cell<br>liver | 1 × 10$^5$ | 193 | 16458 |
| 2 | 62 | F | Renal cell<br>liver, lung | 1 × 10$^5$ | 2297 | 2735 |
| 3 | 57 | F | Hepatoma<br>lung | 1 × 10$^5$ | 105 | 2935 |
| 4 | 34 | F | Colon, lung<br>kidney<br>peritoneum | 2 × 10$^5$ | 364 | 585 |
| 5 | 54 | F | Renal cell<br>lung | 2 × 10$^5$ | 2093 | 27022 |
| 6 | 73 | M | Colon<br>lung, liver | 2 × 10$^5$ | 82 | 2211 |
| 7 | 25 | M | Colon<br>liver | 2 × 10$^5$ | 727 | 3151 |
| 8 | 49 | F | Lung<br>contralateral<br>lung | 2 × 10$^5$ | 115 | 11079 |
| 9 | 63 | M | Renal cell<br>lung, skeletal | 3 × 10$^5$ | 270 | 38274 |

* Top line, primary cancer; below, site of metastatic involvement.

**Table 2.** Clinical/laboratory profile of patients who received 5 day continuous intravenous infusion of rh-TNF

| No. | Age | Sex | Diagnosis* | Dose of rh-TNF U/m$^2$/day | PLA$_2$ U/ml pre-TNF | PLA$_2$ U/ml peak during TNF infusion |
|---|---|---|---|---|---|---|
| 1 | 55 | F | Lung<br>skeletal | 0.5 × 10$^5$ | 263 | 2191 |
| 2 | 55 | M | Carcinoid<br>liver | 0.5 × 10$^5$ | 167 | 485 |
| 3 | 54 | F | Skeletal<br>lung, brain | 0.5 × 10$^5$ | 158 | 8676 |
| 4 | 37 | F | Ovarian<br>liver | 1.0 × 10$^5$ | 106 | 2505 |
| 5 | 49 | F | Breast<br>liver | 1.0 × 10$^5$ | 178 | 9801 |
| 6 | 55 | M | Colon<br>liver | 1.0 × 10$^5$ | 6397 | 13045 |
| 7 | 45 | M | Renal<br>pancreas | 2.0 × 10$^5$ | 601 | 35056 |
| 8 | 48 | M | Colon | 2.0 × 10$^5$ | 264 | 25861 |
| 9 | 58 | F | Colon, pleura<br>liver | 2.0 × 10$^5$ | 265 | 7496 |
| 10 | 67 | F | Uveal<br>melanoma<br>lung | 2.4 × 10$^5$ | 311 | 18038 |
| 11 | 70 | M | Colon | 2.4 × 10$^5$ | 604 | 8950 |
| 12 | 68 | M | Lung | 2.4 × 10$^5$ | 287 | 23163 |
| 13 | 56 | F | Colon<br>mesenteric | 3.0 × 10$^5$ | 224 | 5596 |
| 14 | 58 | M | Renal<br>peritoneum | 3.0 × 10$^5$ | 1984 | 8311 |

* Top line, primary cancer; below, site of metastatic involvement.

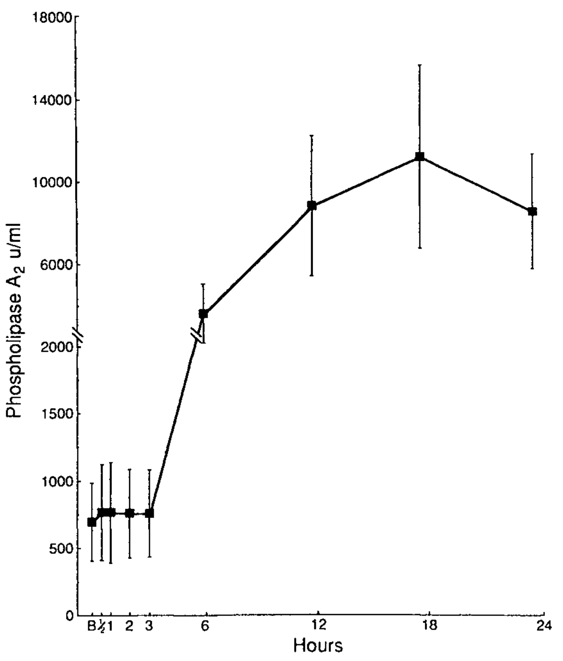

FIG. 1. Phospholipase $A_2$ in patients who received 24 h continuous intravenous infusion of rh-TNF. Vertical bars, mean $\pm$ SEM.

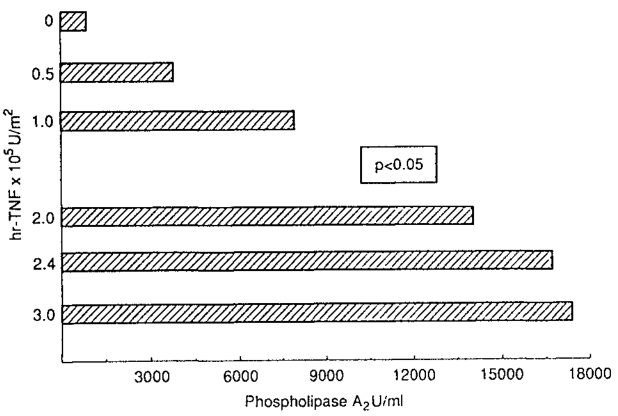

FIG. 3. Correlation between the dose of infused human recombinant TNF and increase of phospholipase $A_2$. Horizontal bars, means.

tralized (93%) $PLA_2$ activity in the sera of rh-TNF infused patients. rh-TNF or etoposide had no detectable effect on the activity of purified $PLA_2$ *in vitro.*

### Discussion

Extracellular phospholipase $A_2$ ($PLA_2$) has recently been identified in synovial fluids in inflammatory arthritides.[3] Subsequently, very high serum activity of $PLA_2$ was found in systemic inflammatory processes such as septic shock[5,6] and adult respiratory distress syndrome.[6] Purified $PLA_2$ instilled into the lungs[21] or injected into joints,[8] skin[7] or paws[9] of experimental animals causes marked dose and time-dependent inflammatory reactions that are abolished by inhibitors of this enzyme. Taken together, these results suggest that $PLA_2$ plays an important role in local and systemic inflammatory processes.[22,23]

Marked increases in circulating $PLA_2$ have been observed in animals challenged with endotoxin.[5] In such animals, the rise in the serum $PLA_2$ activity correlates with the fall in the mean arterial blood pressure. When the $PLA_2$-enriched fraction of septic shock serum is infused into healthy rabbits, it reproduces the clinical and haemodynamic changes induced by endotoxin. Pretreatment of the $PLA_2$-enriched fraction by the $PLA_2$ inhibitor, *p*-bromophenacyl bromide, inhibits $PLA_2$ activity and abrogates the hypotensive effect.[5]

Very high circulating $PLA_2$ activity has been found in patients with gram-negative septic shock.[6] In both retrospective[6] and prospective[24] studies, the activity of $PLA_2$ correlates with the Haemodynamic Instability Score ($p < 0.001$). The above studies have shown that $PLA_2$ fulfils several of Lefer's criteria[25] for a mediator of circulatory shock: (1) a marked increase in circulatory $PLA_2$ in response to bacteria or their toxins; (2) hypotension caused by $PLA_2$ in experimental animals; and (3) correlation of endogenous $PLA_2$ levels with the severity of hypotension in both animals and man. Furthermore, an inhibitor of $PLA_2$, *p*-bromophenacyl bromide, ameliorates these effects of $PLA_2$. Taken together, these data suggest that $PLA_2$ is one of the mediators of septic shock manifestations.

Several studies have recently examined the relationship between $PLA_2$ and cytokines. TNF

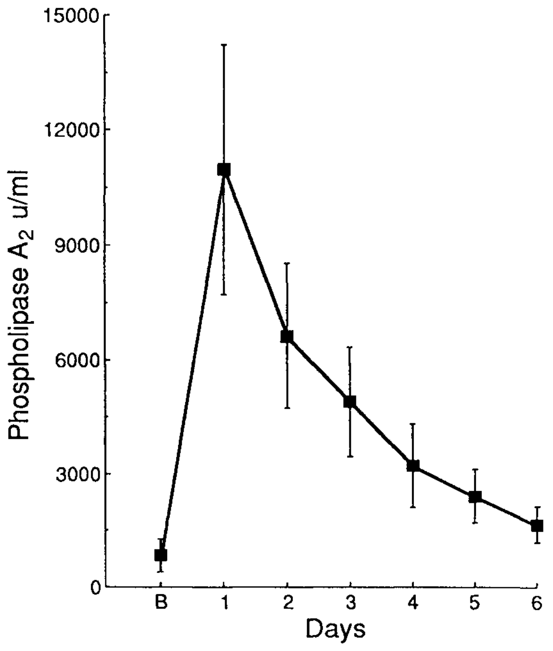

FIG. 2. Phospholipase $A_2$ in patients who received 5 day continuous intravenous infusion of rh-TNF. Vertical bars, mean $\pm$ SEM.

exerts its impact on the cells through its binding to cell surface receptors.[26] The interaction of TNF with the receptors is associated with GTP binding and increase in GTPase activity.[27] Furthermore, the association of TNF and $PLA_2$ has been reported by several groups.[28–33] TNF stimulates $PLA_2$ activity in HL-60[32] and Balb/c 3T3 cells[30] and, conversely, $PLA_2$ activity is required for the transcriptional activation of TNF gene expression.[32,33] Moreover, inhibitors of $PLA_2$ have been found to interfere with the cytotoxic and cytolytic activity of TNF[28,29] and to block TNF-induced increases in macrophage-specific colony stimulating factor (M-CSF) transcripts.[31]

In human volunteers challenged intravenously with endotoxin, marked increases in circulating $PLA_2$ followed transient increases in TNF.[10] This temporal relationship is of particular interest, since it has been found that bacterial or toxin challenge in animals or in man leads to a prompt release of cytokines such as IL-1 and TNF. The ensuing clinical and haemodynamic changes, which in the past were assumed to be related to bacterial toxins, have recently been linked to the effects of cytokines. TNF is capable of eliciting most, if not all, effects of endotoxin.[11,12] It was therefore concluded that TNF is one of the endogenous mediators of endotoxic shock.[11,13,14] Recent studies have also suggested that several physiological and metabolic changes that are associated with malignant processes are in fact mediated through TNF.[18]

Several clinical trials with intravenous administration of rh-TNF have been performed in cancer patients.[16,17,34] The cascade of events following TNF infusions has not been elucidated. Transient increases in circulating IL-6 have been observed in cancer patients infused with TNF. The highest level of IL-6 was observed after 3–6 h of TNF infusion, and correlated with TNF dose.[35] We hypothesized that TNF infusion will also lead to increased $PLA_2$ activity. This postulate was based on the observations that endotoxin infusion leads to increases in TNF followed by high circulating $PLA_2$[10] and that TNF enhances $PLA_2$ synthesis and secretion *in vitro*.[15] Many of the effects of TNF depend on the local and systemic activation of the cyclooxygenase pathway.[13] Furthermore, prostaglandins appear to play a role in mediating the effects of TNF.[14] Thus, if the activity of $PLA_2$ is modulated by TNF, the role of eicosanoids in proximal cytokine mediated reactions would become more apparent.

In the present study we have shown that in 21 of 23 patients infusions of rh-TNF were associated with significant increases in the activity of circulating $PLA_2$. The earliest serum $PLA_2$ increases occurred 3 h after beginning the rh-TNF infusion and maximal levels were observed at 18–24 h. Of interest is the fact that in the patients who received 5 day rh-TNF infusion, the level of circulating $PLA_2$ declined after the first 24 h. This phenomenon may be related to the previously described decline in TNF levels that occurs in patients who received 24 h continuous infusions.[16] Neither the relationship between the saturation of TNF receptors and its impact on $PLA_2$ release, nor the intra-vascular/extravascular distribution of $PLA_2$ or its metabolism are known. However, the maximal increase in $PLA_2$ activity correlated with the daily dose of rh-TNF. Therefore a dose-related link was established between TNF and $PLA_2$ activation. Thus, $PLA_2$ is activated by both endotoxin[5,10] and by proximal cytokines. Since $PLA_2$ is proinflammatory and vasoactive, some manifestations previously attributed to endotoxin and tumour necrosis factor should probably be attributed to $PLA_2$ as well.

### References

1. Verheij HM, Slotboom AJ, de Haas GH. Structure and function of phospholipase $A_2$. *Rev Physiol Biochem Pharmacol* 1981; **91:** 91–203.
2. Trang LE. Prostaglandins and inflammation. *Semin Arthr Rheum* 1980; **9:** 153–190.
3. Pruzanski W, Vadas P. Secretory synovial fluid phospholipase $A_2$ and its role in the pathogenesis of inflammation in arthritis. *J. Rheumatol* 1988; **15:** 1601–1603.
4. Vadas P, Pruzanski W, Stefanski E, Johnson L, Seilhamer J, Mustard R, Bohnen J. Phospholipases $A_2$ in acute bacterial peritonitis in man. In: Dennis EA, Hunter T and Berridge M, eds. *Cell activation and signal initiation: receptor and phospholipase control of inositol phosphate, PAF, and eicosanoids production.* New York: Alan Liss Inc., 1989; 311–316.
5. Vadas P, Hay JB. Involvement of circulating phospholipase $A_2$ in the pathogenesis of the hemodynamic changes in endotoxin shock. *Can J Physiol Pharmacol* 1983; **61:** 561–566.
6. Vadas P, Pruzanski W, Stefanski E, Sternby B, Mustard R, Bohnen J, Fraser J, Farewell V, Bombardier C. Pathogenesis of hypotension in septic shock: correlation of circulating phospholipase $A_2$ levels with circulatory collapse. *Crit Care Med* 1988; **16:** 1–7.
7. Pruzanski W, Vadas P, Fornasier V. Inflammatory effect of intradermal administration of soluble phospholipase $A_2$ in rabbits. *J Invest Dermatol* 1989; **86:** 380–383.
8. Vadas P, Pruzanski W, Kim J, Fornasier V. The proinflammatory effect of intraarticular injections of soluble human and venom phospholipase $A_2$. *Am J Pathol* 1989; **134:** 807–811.
9. Vishwanath BS, Fawzy AA, Franson RC. Edema-inducing activity of phospholipase $A_2$ purified from human synovial fluid and inhibition by aristolochic acid. *Inflammation* 1988; **12:** 549–561.
10. Pruzanski W, Stefanski E, Wilmore DW, Martich GC, Hoffman AGD, Suffredini A, Vadas P. Sequential activation of TNF-phospholipase $A_2$ axis following i.v. endotoxin challenge in human volunteers. *FASEB J* 1990; **4:**A 1714.
11. Beutler B, Cerami A. The endogenous mediator of endotoxic shock. *Clin Res* 1987; **34:** 192–197.
12. Tracey KJ, Lowry SF, Cerami A. Cachectin: A hormone that triggers acute shock and chronic cachexia. *J Infect Dis* 1988; **157:** 413–420.
13. Michie HR, Guillou PJ, Wilmore DW. Tumor necrosis factor and bacterial sepsis. *Br J Surg* 1989; **76:** 670–671.
14. Simpson SQ, Casey LC. Role of tumour necrosis factor in sepsis and acute lung injury. *Crit Care Clin* 1989; **5:** 27–47.
15. Vadas P, Pruzanski W, Stefanski E, Ellies L, Aubin J, Sos A, Melcher A. Extracellular phospholipase $A_2$ secretion is a common effector pathway of interleukin-1 and tumour necrosis factor action. *Immunol Lett* 1991; **28:** 187–194.
16. Spriggs DR, Sherman ML, Michie H, Arthur KA, Imamura K, Wilmore D, Frei III E, Kufe DW. Recombinant human tumour necrosis factor administered as a 24 hour intravenous infusion. A phase 1 and pharmacologic study. *J Natl Canc Inst* 1988; **80:** 1039–1044.
17. Sherman ML, Spriggs DR, Arthur KA, Imamura K, Frei III E, Kufe DW. Recombinant human tumour necrosis factor administered as a five day continuous infusion in cancer patients: phase I toxicity and effects on lipid metabolism. *J Clin Oncol* 1988; **6:** 344–350.
18. Michie HR, Sherman ML, Spriggs DR, Rounfs J, Christie M, Wilmore DW. Chronic TNF infusion causes anorexia but not accelerated nitrogen loss. *Ann Surg* 1989; **209:** 19–24.

19. Stefanski E, Pruzanski W, Sternby B, Vadas P. Purification of soluble phospholipase $A_2$ from synovial fluid in rheumatoid arthritis. *J Biochem* 1986; **100:** 1297–1303.
20. Vadas P, Pruzanski W, Stefanski E, Sternby B. Compartmental heterogeneity of soluble phospholipases A. *Inflammation* 1990; **14:** 173–183.
21. Edelson JD, Vadas P, Villar J, Mullen JBM, Pruzanski W. Acute lung injury induced by phospholipase $A_2$. Structural and functional changes. *Am Rev Resp Dis* 1991; **143:** 1102–1109.
22. Pruzanski W, Vadas P. Phospholipase $A_2$—a mediator between proximal and distal effectors of inflammation. *Immunol Today* 1991; **12:** 143–146.
23. Vadas P, Wasi S, Movat HZ, Hay JB. Extracellular phospholipase $A_2$ mediates inflammatory hyperemia. *Nature* 1981; **293:** 583–585.
24. Vadas P, Pruzanski W, Stefanski E, Ruse J, Farewell V, McLaughlin J, Bombardier C. Concordance of endogenous cortisol and phospholipases $A_2$ in gram negative septic shock: a prospective study. *J Lab Clin Med* 1988; **111:** 584–590.
25. Lefer AM. Eicosanoids as mediators of ischaemia and shock. *Fed Proc* 1985; **44:** 275–280.
26. Larrick JW, Kunkel SL. The role of tumour necrosis factor and interleukin 1 in the immunoinflammatory reponse. *Pharma Res* 1988; **5:** 129–139.
27. Imamura K, Sherman ML, Spriggs D, Kufe D. Effect of tumour necrosis factor on GTP binding and GTPase activity in HL-60 and L929 cells. *J Biol Chem* 1988; **263:** 10247–10253.
28. Suffys P, Beyaert R, Van Roy F, Fiers W. Reduced tumour necrosis factor-induced cytotoxicity by inhibitors of arachidonic acid metabolism. *Biochem Biophys Res Commun* 1987; **149:** 735–743.
29. Neale ML, Fiera RA, Matthews N. Involvement of phospholipase $A_2$ activation in tumour cell-killing by tumour necrosis factor. *Immunology* 1988; **64:** 81–85.
30. Palombella VJ, Vilcek J. Mitogenic and cytotoxic actions of tumour necrosis factor in BALB/c 3T3 cells. *J Biol Chem* 1989; **264:** 18128–18136.
31. Sherman ML, Weber BL, Datta R, Kufe W. Transcriptional and post-transcriptional regulation of macrophage-specific colony stimulating factor gene expression by tumour necrosis factor. *J Clin Invest* 1990; **85:** 442–447.
32. Spriggs DR, Sherman ML, Imamura K, Mohri M, Rodriguez C, Robbins G, Kufe DW. Phospholipase $A_2$ activation and autoinduction of tumour necrosis factor gene expression by tumour necrosis factor. *Cancer Res* 1990; **50:** 7101–7107.
33. Mohri M, Spriggs DR, Kufe D. Effects of lipopolysaccharide on phospholipase $A_2$ activity and tumour necrosis factor expression in HL-60 cells. *J Immunol* 1990; **144:** 2678–2682.
34. Demetri GD, Spriggs DR, Sherman ML, Arthur KA, Imamura K, Kufe DW. A phase I trial of recombinant human tumour necrosis factor and interferon-gamma: effects of combination cytokine administration *in vivo*. *J Clin Oncol* 1989; **7:** 1545–1553.
35. Brouckaert P, Spriggs DR, Demetri G, Kufe DW, Fiers W. Circulating interleukin 6 during a continuous infusion of tumour necrosis factor and interferon. *J Exp Med* 1989; **169:** 2257–2262.

ACKNOWLEDGEMENTS. This work was supported by grants-in-aid from the Medical Research Council of Canada and the Arthritis Society, National Institutes of Health Grant CA 42802 and by a Burroughs Wellcome Award in Clinical Pharmacology (DWK).

**Received 31 March 1992; accepted in revised form 11 May 1992**
